# What a mix! Volatile organic compounds and worker exposure in small business beauty salons in Tucson, Arizona

**DOI:** 10.3389/fpubh.2023.1300291

**Published:** 2023-12-18

**Authors:** Denise Moreno Ramírez, Shannon Gutenkunst, Nathan Lothrop, Carolina Quijada, Marvin Chaires, Imelda Cortez, Flor Sandoval, Fernanda J. Camargo, Emma V. Gallardo, Elmira Torabzadeh, Rietta Wagoner, Nicolas Lopez-Galvez, Maia Ingram, Dean Billheimer, Ann Marie Wolf, Paloma I. Beamer

**Affiliations:** ^1^Mel and Enid Zuckerman College of Public Health, The University of Arizona, Tucson, AZ, United States; ^2^BIO5 Institute, The University of Arizona, Tucson, AZ, United States; ^3^Sonora Environmental Research Institute, Inc., Tucson, AZ, United States; ^4^College of Health and Human Services School of Public Health, San Diego State University, San Diego, CA, United States

**Keywords:** VOC exposure, beauty salons, hierarchy of controls, salon products, worker health, Spanish-speaking small businesses, heat-styling, beauty justice

## Abstract

**Introduction:**

Small business beauty salons have volatile organic compounds (VOCs) in their workplace air. VOCs are present as ingredients in beauty or hair products. They may also form because of chemical reactions, where thermal-styling elements accelerate the volatilization of these compounds. Uncertainties remain about the relationship between air pollutant concentrations and the variety of beauty salon activities in a work shift. Investigating these associations can help determine high-risk services, associated products, and at-risk workers.

**Methods:**

In this exploratory study, female community health workers recruited beauty salons from target zip codes in predominately Latino neighborhoods, including primarily Spanish-speaking small businesses. We collected salon chemical inventories, business characteristics, and participant activity logs to understand how chemicals and activities influence the total and specific VOC concentrations. We sampled personal total VOCs and specific VOCs from the same shop during the participant work shift. We also measured personal total VOCs for four work shifts per shop.

**Results:**

A linear mixed effects model of log VOCs on the fixed effect of activity and the random effects of salon and shift within the salon showed that the variance between salons explains over half (55%) of the total variance and is 4.1 times bigger than for shifts within salons. Summa canisters detected 31 specific VOCs, and hazard scores ranged between 0 and 4.3. 2-Propanol (isopropyl alcohol) was the only VOC detected in all shifts of all salons.

**Discussion:**

In this study, differences in VOC measurements were primarily between salons. These differences may result from differences in ventilation, services rendered, and product lines applied.

## 1 Introduction

Beauty salons are ubiquitous. This industry is estimated to be valued at $230.64 billion worldwide, with profits increasing to $383.88 billion by 2030 (1). In the United States (U.S.), estimates suggest that in 2023, individuals will spend about $91.23 billion on hair care products alone (2). Currently, in the U.S., the beauty industry is expanding at a high rate, spurred by a general trend toward wellness (3). The beauty market has also demonstrated resilience in turbulent macroeconomic times because of the coronavirus pandemic, making it a lucrative investment for many entrepreneurs, celebrities, and influencers (4, 5). Yet, since the early twentieth century, the production of cosmetics has been dominated by a handful of multinational corporations with significant influence over the type and content of products that workers and consumers may be exposed to in beauty salons (6).

Air in beauty salons contains volatile organic compounds (VOCs) that may harm human health. These compounds are introduced through ingredients in beauty and hair products used to provide clients with the desired style. They may also form because of chemical reactions during the styling process. Often, volatilization of these compounds is accelerated by thermal hair-styling tools (e.g., hairdryers, flatirons, hot combs, and hair processors) used in hair-styling, processing, and cutting activities completed daily in salons. Thermal hair-styling tools can use different technologies ranging from ceramic to ionic and infrared to tourmaline. Typically, a hair dryer heats the air surrounding wet strands of hair, and as the capacity of the surrounding air to hold moisture increases, the water from the hair evaporates. In the case of ionic hairdryers, they contain a negative ion-generating device that helps smooth frizz. Other VOC sources in a beauty salon may include disinfection products and other environmental sources (e.g., traffic-related air pollution). Consequently, beauty salon workers and their clients are exposed to salon VOCs through their products and activities. Yet, salon workers experience more frequent and prolonged exposure to these compounds.

Beauty salons are a significant employer of women in the U.S. Most small businesses (less than 100 employees) fall under the category of the professional services sector (7). Estimates suggest approximately 80,000 beauty salons exist in the U.S. (8). Businesses with less than 20 workers employ 21 million workers, and over 20 million are employed by firms with 20–99 workers, representing about 17% of the worker population (9). Small business-sized beauty salons mostly employ racial and ethnic minority workers who often have gaps in health insurance coverage and suffer disproportionate health impacts (10).

Previous investigations on VOCs focus primarily on nail salons, yet beauty salons provide more services and are understudied. Exposures in beauty salon settings are varied and potentially even higher. Also, more adverse health outcomes are associated with performing hair processes than nail care tasks (11). Current research on beauty salons has focused on beautician exposure via biomonitoring, identifying compounds in workplace air, and how compounds interact under controlled experimental conditions (12–16). This existing research concludes by raising health concerns for beauty salon workers. One study found that workers serving primarily clientele of color have VOC concentrations in their bodies approximately four times higher than those of general women in the U.S. (16).

Understanding the relationship between air pollutant concentrations and the variety of cosmetic practices that occur throughout the work shift of a beauty salon worker is vital to developing strategies to protect worker health. Investigating these associations can help determine high-risk services, associated products, and at-risk workers. The application of controls has also been understudied in the beauty salon setting. From occupational health studies, we know the hierarchy of controls (HoC) can assist in selecting safeguards to protect worker health. More information is needed on the feasibility of safety protections and how to facilitate their implementation in an overburdened setting with esthetics as a priority. Identifying controls is especially important because many of the chemicals found in beauty salons have been shown to impact workers’ reproductive systems, lung functions, cardiovascular system, cognition, and skin health (11, 17, 18). Additionally, the health impact on these workers may only be diagnosed decades after the exposure, and associations are underrecognized and underreported in occupational surveillance data (19–21). Even with the potential risk to workers and surrounding communities, few studies have quantified VOC exposures in small businesses, and most are out of date, limited in scope, or conducted outside the U.S.

The current project evolved from grassroots pollution prevention work established by Mexican community health workers (CHWs) employed by the Sonoran Environmental Research Institute, Inc. (22). CHWs are frontline public health workers who identify strongly with their communities, and they have a long history of addressing environmental health concerns (23). In this exploratory study, we sought to determine how different beauty salon activities influence workplace air compounds and whether these compounds vary more between salons than within a salon. We measured total VOCs as our primary outcome because methods that measure specific VOCs (our secondary outcome) cannot account for all the VOCs. Also, elucidating a whole class of chemicals that can be targeted with a system-wide intervention rather than one chemical at a time is more efficient. The information gained from this study will be utilized to reduce beauty salon workplace exposures to VOCs. A follow-up study aims to understand if an industrial-hygiene-enhanced CHW intervention can minimize exposure to VOCs in the workplace of beauty salons and auto shop small businesses. We concentrate our efforts on small beauty salon businesses in southern metropolitan Tucson, Arizona, with a high population identifying as Mexican and Mexican American (Latino).

## 2 Materials and methods

### 2.1 Study population and recruitment

The Solutions for a Changing World Project brings together the Sonora Environmental Research Institute, Inc. (SERI), El Rio Community Health Center (federally qualified health center), and the University of Arizona’s (UA) Mel and Enid Zuckerman College of Public Health (MEZCOPH). We implemented an exploratory study to characterize the compounds in workplace air in 10 small business beauty salons in 2018. This study defines small businesses as those with 25 employees or fewer.

During the study period, we primarily focused on developing partnerships with small businesses and assessing salon exposures and activities in four work shifts. We collected total and specific VOC samples, business characteristics (e.g., ventilation conditions and number of rooms), participant demographics, participant activity logs (e.g., salon activities, activity start and end times), and salon chemical inventories. The data collection period was from June until November 2018.

The City of Tucson is situated in the semi-arid Sonoran Desert in southern Arizona and has one of the country’s highest poverty rates (24). CHW recruited beauty salons from six target ZIP codes. We focus on these ZIP codes because those who live in them have higher poverty rates, increased urban stress, and lower educational attainment. One of the nation’s oldest Superfund sites is in this area (Tucson International Airport Area). The ZIP codes also contain Tucson’s Latino neighborhoods and predominately Spanish-speaking small businesses. The beauty salons located here also primarily serve Latino clients.

CHW from SERI recruited participants from small businesses beauty salons in person or via phone. Some businesses were approached because of their previous interaction with SERI in a pollution prevention program. The remaining businesses were approached because they existed in a SERI database. When business owners agreed to participate in the study, the CHW obtained written permission and consent from individual workers at the salon participating in the study. Workers had to be consented separately. The business owner had to consent for the business to participate in the study, but not all workers (including the owner) at the business were required to participate in personal total VOC monitoring. Demographic information was acquired from participants.

Inclusion criteria for this study comprise being a small business beauty salon in the selected ZIP codes (25 employees or less), an owner, manager, or employee who is at least 18 years old, able to speak Spanish or English, and expected to be employed at the business for the next 3 months. Exclusion criteria include nail salons and chain beauty salon businesses, and businesses outside the targeted ZIP codes. Study subjects were not compensated for participation but would receive results from the air monitoring events and received consultation with a health insurance navigator. The UA Human Subjects Protection Program approved all human-subjects materials related to the study (#1709821542).

Salon chemical inventories, business characteristics, and participant activity logs were recorded throughout the work shifts to understand how chemicals and activities influence the total and specific VOC concentrations. Public health researchers from the U.A. were embedded in the field, observing salon activities and business characteristics (e.g., ventilation) while monitoring a work shift. Notes were taken in a form designed for field observations. The categories developed for beautician activities were: (1) administration; (2) clean-up/housekeeping; (3) hair processing; (4) hair-styling/cutting; (5) skin care; (6) taking a break; and (7) unknown. These categories were identified as the most general activities that could occur in a salon. The participant activity log also included ventilation categories: (1) central air conditioning; (2) swamp cooler; (3) mini-split/wall air conditioning unit; (4) desk/floor fan; (5) ceiling fan; (6) open door/window; and (7) local exhaust fan, as well as other business conditions such as the number of rooms present at the business. The activity log also captured the specific products applied and issues with monitoring equipment.

### 2.2 VOC measurements – total and specific VOCs

Total VOC measurements were collected during four work shifts per salon. Total VOCs were measured using real-time photoionization detectors (PIDs) ppbRAE 3000 (RAE Systems, Inc. San Jose, CA). The PID monitor was placed on the individual in a specific bag slung on their shoulder, belt, or near them as they performed the salon activity. A Versilon SE-200 fluorinated ethylene-propylene lined tubing (Saint-Gobain, Courbevoie, France), with one end connected to the monitor and the other end placed near the participant’s face, was used to measure total VOCs measured closest to the participant’s breathing zone. Each monitor was set to record total VOCs every 20 s. Public health researchers addressed issues resulting from participants’ monitoring equipment during their work shifts by viewing a handheld EchoView Host (RAE Systems, Inc., San Jose, CA). Detailed methodological steps are reported by Lothrop and colleagues (25).

Specific VOC samples were collected during one or two salon visits, with half of the salons having Summa canister (Restek^™^ SilcoCan Air Canisters with RAVE Valve) measurements on two separate days and one salon having a true duplicate on the same day. Summa canisters were put in the room with the most expected activity on the floor in a location that would not interrupt workflow. Sample preparation, analyte determination, and measurement methods for specific VOCs followed the U.S. Environmental Protection Agency (U.S. EPA) Air Method Toxic Organics-15, which tests for 70+ VOCs (26). Test America Laboratories, Inc. completed laboratory analyses and tentatively identified additional VOCs beyond the standard set for TO-15.

### 2.3 Data analysis – total VOCs

Values of total VOCs below the limit of detection (LOD) and recorded as 0 parts per billion (ppb) by the PID were replaced with LOD/√2 = 1 ppb/√2 ≈ 0.707 ppb before proceeding with the statistical analysis. Additionally, observations where the ppbRAE was left running in the salon but the participant left the salon were identified by notes in the activity logs and removed. Likewise, before proceeding with further analyses, observations where the ppbRAE was malfunctioning were identified in the notes as a flow fault or ppbRAE alarm in the activity logs, and then suspicious data were removed by looking at the data (mainly VOC concentrations below the LOD, but at least below the baseline level for the shop). A flow fault could occur from the tubing leading from the monitor to near the participant’s face becoming crimped. When a U.A. public health researcher noticed such a flow fault, they would fix it (allowing sampling to resume) and note the time of the fix in the activity log.

Because the total VOC data were correlated in time, we used the aggregated data over each activity-ventilation span (whenever the activity or ventilation changed): each observation was the average (arithmetic mean) for each sequential activity-ventilation span during each shift. Because all total VOC measurement intervals were equal lengths (20 s), this average could be considered a time-weighted average. Because the distribution of this aggregated total VOC data was skewed, the data were log-transformed before statistical analysis was completed.

### 2.4 Data analysis – specific VOCs

Summa canister results for the 70+ chemicals attempted to be measured in all the small business beauty salons (following U.S. EPA Air Method Toxic Organics – 15) and detected in at least one salon were plotted and used in this analysis. Given the complexity of the mixtures in each of the salons and to allow comparisons between salons we estimated a hazard score for the mixture. The hazard score was determined by calculating the measured VOC concentration in ppb divided by that VOC’s reference concentration in ppb and summing that quantity for all measured VOCs in that Summa canister in a salon on a given date. U.S. EPA’s inhalation reference concentration (RfC) values were used as the reference concentration because they were available for more chemicals in this study than ACGIH TLV (see Supplementary material).

If a chemical from the Summa canister result did not have a given U.S. EPA Inhalation RfC value (in units of micrograms per cubic meter or μg/m^3^), then an estimated inhalation reference value was calculated using the inhalation cancer unit risk factor (IUR) of the chemical. The IUR was converted to the desired μg/m^3^ units using the following conversion:

$$RfC_{\mathrm{fromIUR}}=\left(10^{-4}\right)/\left(IUR\right)$$

The 10^−4^ value is used to be consistent with Hazardous Air Pollutants (HAP) guidelines for cancer risk assessment. If the chemical from the Summa canister did not have a U.S. EPA inhalation RfC value nor an IUR value, an assumed inhalation reference value was produced using the reference oral dose (RfD in mg/(kg * day)) of the chemicals. This assumed inhalation value was converted to inhalation units in μg/m^3^ with the following equation:

$$\begin{array}{c} RfC_{\mathrm{fromRfD}}\;\left(\mathrm{\mu g}/m^{3}\right)=RfD\;\left(\mathrm{mg}/\left(\mathrm{kg}*\mathrm{day}\right)\right)\;\\ x\;\left(70\;\mathrm{kg}/20\;m^{3}/\mathrm{day}\right)\;x\;1000\;\mathrm{\mu g}/\mathrm{mg} \end{array}$$

The assumptions for the conversion were 70 kilograms as the average adult body weight and the average daily adult inhalation rate of 20 m^3^ per day (both values were derived from the U.S. EPA Exposure Factors Handbook). For benzyl chloride, the U.S. EPA cancer oral slope factor (in units of risk per mg/(kg * day)) to μg/m^3^ was used to produce an assumed inhalation reference value.

$$\begin{array}{l} RfC_{\mathrm{benzylchloride}}\;\left(\mathrm{\mu g}/m^{3}\right)=[\left(10^{-4}\right)\\ /(\begin{array}{l} \mathrm{cancer}\;\mathrm{oral}\;\mathrm{slope}\;\mathrm{factor}\;\mathrm{in}\;\mathrm{risk}\;\mathrm{per}\;\mathrm{mg}\\ /\mathrm{kg}-\mathrm{day} \end{array}]*1000\;\mathrm{\mu g}/\mathrm{mg} \end{array}$$

After all these assumptions were made, we ended up with 56 number of RfCs which were used in the calculation of the hazard scores (see table at start of Supplementary material for the reference value and its source for each chemical).

### 2.5 Statistical analysis

Data cleaning and statistical analyses were performed using R version 4.3.1, the *tidyverse* package for data manipulation, the *lme4* package for linear mixed effects models, and other packages listed in the Supplementary material (27–29). A value of *p* < 0.05 was assumed to be statistically significant. Descriptive statistics were calculated to characterize the data.

To examine the relationship between total VOCs and beautician activities, salons, and shifts within salons, we fit a linear mixed effects model of log VOCs on the fixed effect of activity and the random effects of salon and shift within salon. Each salon and shift within a salon were allowed to have a different intercept, and shifts were placed within salons in the model to account for their nested structure. Note that a frequency table of observations for salon and activity showed that skincare had limited observations, so this beautician activity was dropped from the mixed effects model.

## 3 Results

### 3.1 Participants

CHWs visited 15 small business beauty salons to recruit the 10 salons participating in this study. From these 10 salons, nine out of 10 (90%) eligible owners and 14 out of 25 (56%) employees consented to personal total VOC sampling. All shops allowed for the monitoring of specific VOC sampling.

Demographic information is provided in Table 1. The 23 beauty shop workers who agreed to wear PIDs were Latino between the ages of 30 and 65, primarily female (21/23 = 91.3%), with a mix of employees (14/23 = 60.9%) and owners (9/23 = 39.1%). Most participants also consented in Spanish (22/23 = 95.7%) or were Spanish speaking. No managers participated in this study.

**Table 1:** Participant demographics for the 23 participants from salons 1–10 with total VOCs data.

	Overall (*N* = 23)
Age	
Missing	3
Mean (SD)	46.5 (9.3)
Range	30.0–65.0
Gender	
Female	21 (91.3%)
Male	2 (8.7%)
Worker type	
Employee	14 (60.9%)
Owner	9 (39.1%)
Ethnicity	
Latino	23 (100.0%)
Consent language	
English	1 (4.3%)
Spanish	22 (95.7%)

### 3.2 Total VOCs

Total VOC samples were collected from 23 participants throughout 40 shifts at 10 beauty salons from the recruitment area using PIDs. Before data cleaning of times when the monitor was left running in the salon, but the participant left the salon, and of times when the monitor malfunctioned, there were 8,903/49,624 = 17.9% of the observations below the LOD; after there were 6,472/45,471 = 14.2% below the LOD. Once the data were aggregated over the activity-ventilation span as described above, 100/1,200 = 8.3% of the observations were below the LOD.

Because the total VOC data were correlated in time, we used aggregated data over the activity-ventilation span. The number of data points during each work shift (1–4) and salon (B001-B010) after aggregation is shown in Table 2.

**Table 2:** The total number of data points after aggregating the data over each activity-ventilation span, for each shift (1–4) in each beauty salon (B001-B010).

Shift/Salon	B001	B002	B003	B004	B005	B006	B007	B008	B009	B010
1	47	8	42	24	29	34	35	21	22	16
2	46	28	46	27	14	9	49	14	19	42
3	39	22	8	42	35	21	23	29	52	35
4	83	27	36	26	15	14	30	25	28	38

After aggregation, the total VOCs ranged from less than the LOD of 1 ppb to a maximum of 76,892 ppb with a geometric mean of 792 ppb and median of 2,169 ppb.

### 3.3 Work activity and total VOCs

**Figure 1:**
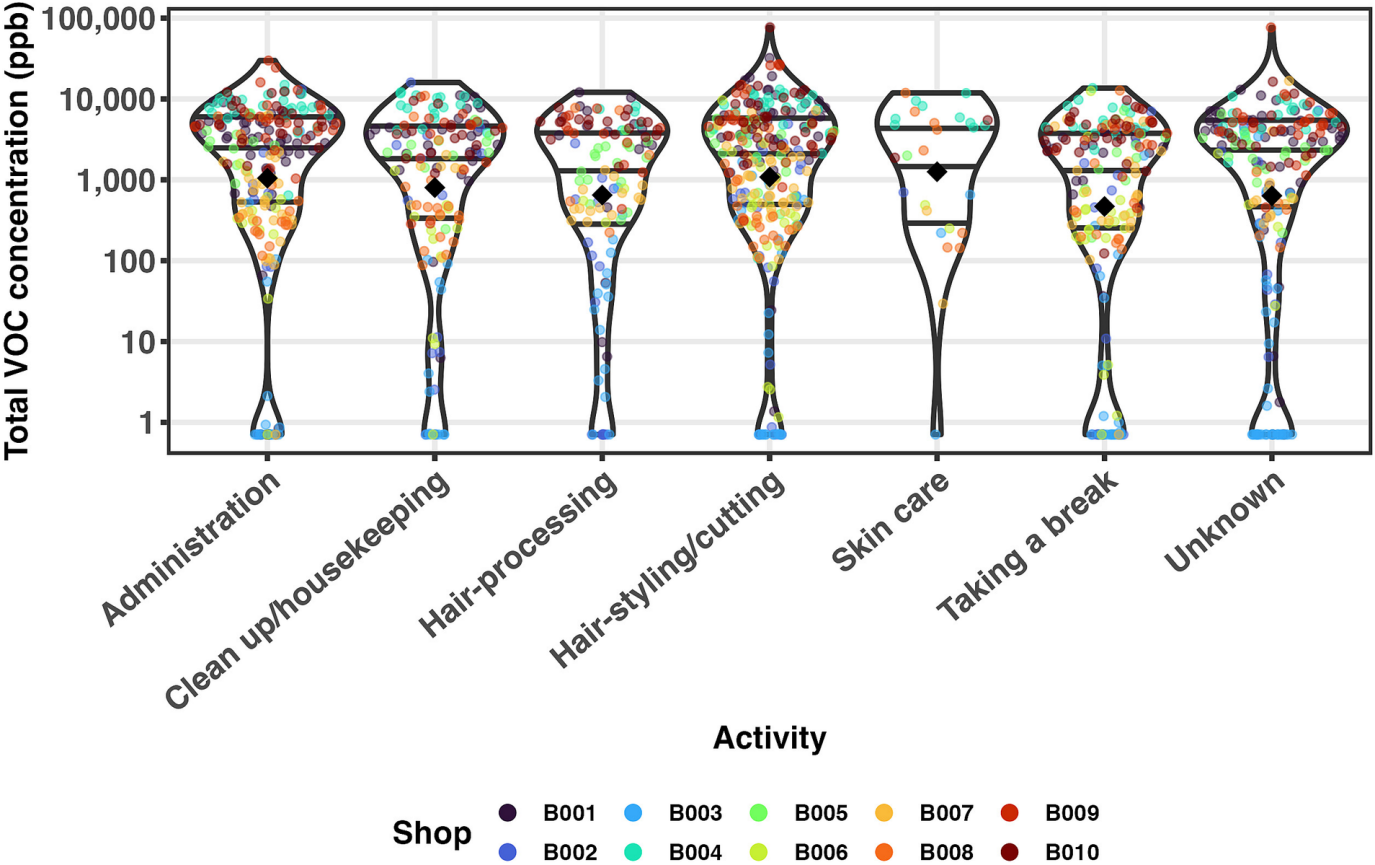
Total VOC concentration grouped by activity and colored by salon. The first quartile (Q1), median, and third quartile (Q3) are shown by black horizontal lines; a black diamond shows the geometric mean. Because of the temporal autocorrelation of the real-time VOC data, each data point represents a time-weighted average for each activity-ventilation span. Due to the skewed distribution of the total VOC concentration, the plot is on a log scale.

To examine the relationship between work activity and total VOCs, Figure 1 shows the distribution of total VOC exposure aggregated over each activity-ventilation span for each salon, as grouped by activity. Datapoints from different salons create the two-peaked structure, with certain salons (B001, B004, B005, B009, B010) dominating contributions to the peak at higher VOCs (between 1,000 and 10,000) and others (B006, B007, B008) dominating contributions to the peak below (between 100 and 1,000 ppb), regardless of activity. Salon B002 and B003 contribute to both peaks and the lower peak, respectively. Thus, this figure shows evidence of the association between salon and VOC exposure. It also shows how small the effect size of activity is compared to that of the salon because it shows only slight (almost unnoticeable) differences in VOC levels between activities.

**Figure 2:**
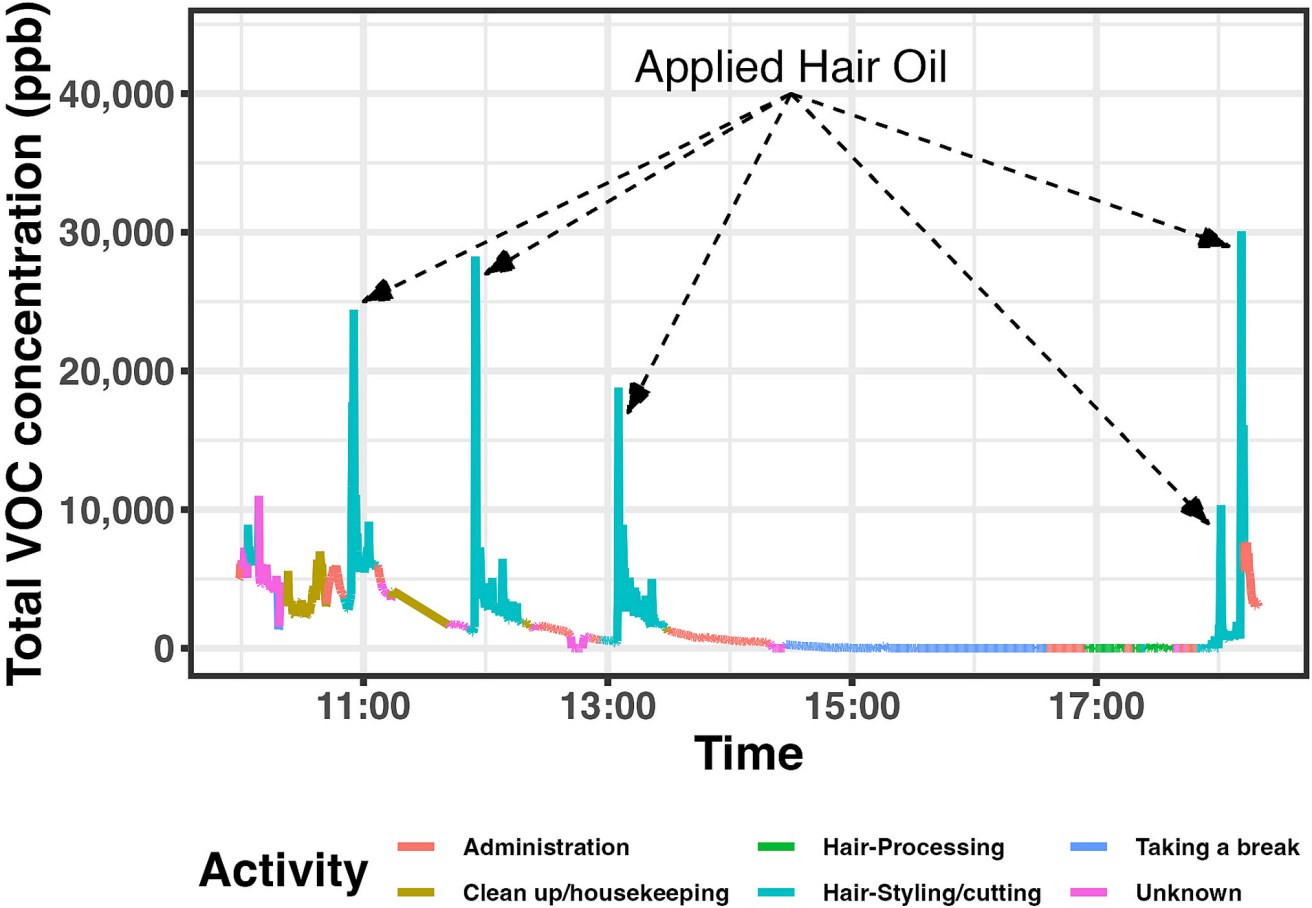
Real-time total VOC data from one shift (B001W01–2018-07-03) with labeled peaks.

Real-time total VOC concentrations are highly variable over the work shift. In the following example, showing real-time toral VOC data from one work shift (Figure 2), peak exposures occurred when hair oil was applied before thermal application during the hair-styling/cutting activity. Furthermore, peak exposures occurred frequently in other shifts when thermal styling occurs after applying hair oil product.

### 3.4 Mixed model

We fit a linear mixed effects model of log total VOC concentration on the fixed effect of activity and the random effects of salon and shift within the salon. The variance between salons accounts for over half (55%) of the total variance in log total VOC concentration and is 4.1 times bigger than that for shifts within salons (Table 3). This indicates that differences between salons like ventilation or beauty product lines contribute more to VOC exposures than specific worker behaviors or activities.

**Table 3:** Variances and standard deviations from the model of log VOCs on the fixed effect of activity and the random effects of salon and shift within salon.

Groups	Variance	Standard deviation	Percent total variance
Shift within salon	1.1	1.0	13
Salon	4.5	2.1	55
Residual	2.6	1.6	32

Analysis of variance shows that activity is significantly associated with the log of the total VOC concentrations (*p* = 0.001 < 0.05; Table 4). However, as noted previously, activity has a small effect size: it does not explain much of the variance in log VOCs.

**Table 4:** Analysis of variance for the model of log VOCs on the fixed effect of activity and the random effects of salon and shift within salon with the Kenward-Roger degrees of freedom method.

	SS	MS	Num df	Den df	*F* value	*p*-value
Activity	53.7	10.7	5	1,136	4.052	0.001

These are Type III sums of squares, which is the sum of squares given all other effects in the model. SS, sum of squares; MS, mean squares; Num df, numerator degrees of freedom; Den df, denominator degrees of freedom.

### 3.5 Specific VOCs

**Figure 3:**
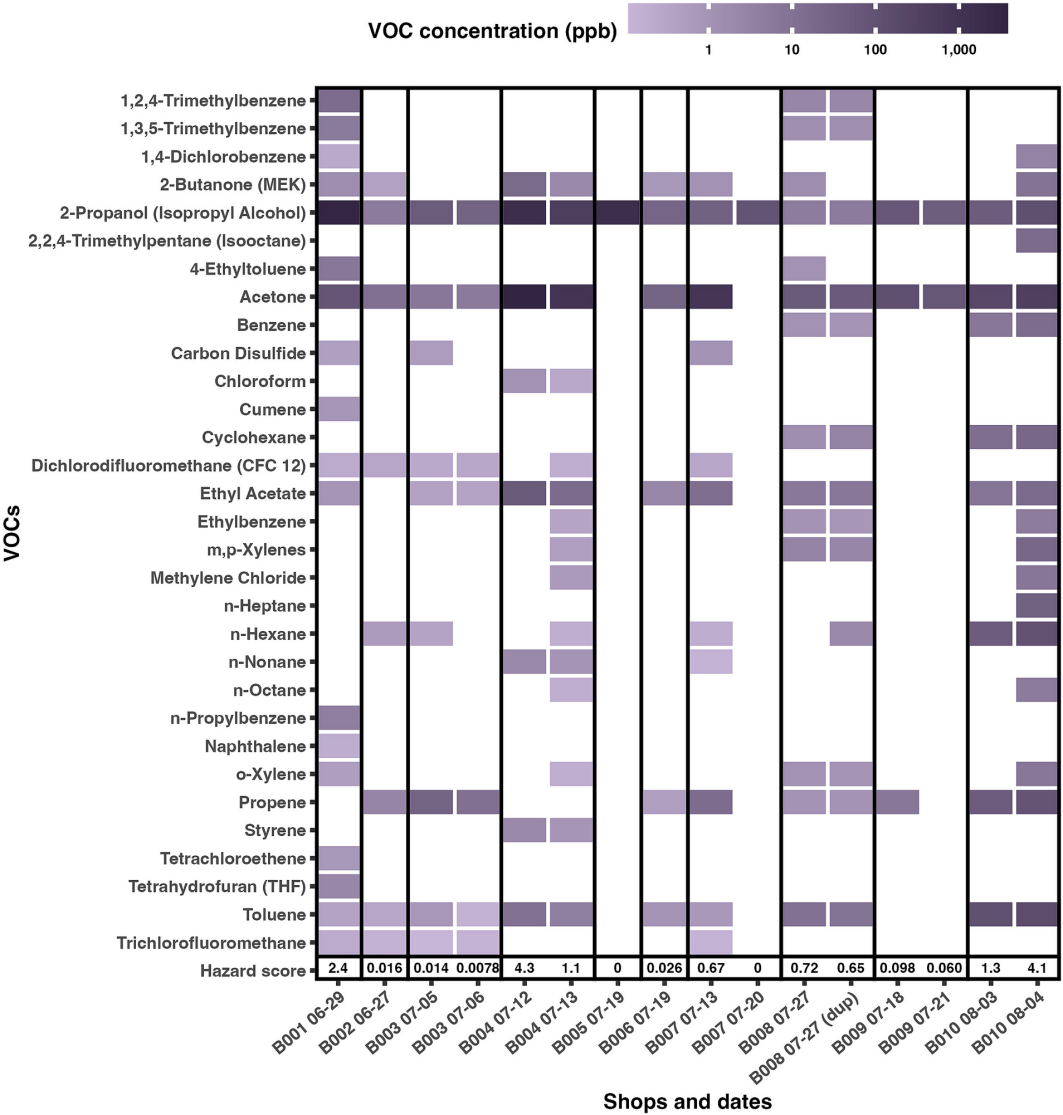
VOC concentration (ppb) of specific VOCs measured by the Summa canisters in 10 beauty salons, for VOCs measured according to the US EPA TO-15 method detected in at least one salon. The hazard score was determined by calculating the measured VOC concentration in ppb divided by that VOC’s reference value in ppb and summing that quantity for all measured VOCs in a Summa canister in a salon on a given date, and it is presented in the last row of the figure.

The Summa canisters detected 31 specific VOCs, and hazard scores ranged between 0 and 4.3 (Figure 3). Hazard scores were similar within a salon, even though different chemicals were detected on different days or even the same day for the duplicate. 2-Propanol (isopropyl alcohol) was the only VOC detected in all Summa canisters in all salons. The other most common chemicals detected were acetone (found in 13/15 canisters and 9/10 shops), toluene (found in 11/15 canisters and 8/10 shops), ethyl acetate (found in 10/15 canisters and 7/10 shops), propene (found in 9/15 canisters and 7/10 shops) and MEK (found in 8/15 canisters and 7/10 shops). The most hazardous chemicals that were found in the salons (based on having low reference values) were naphthalene, chloroform, tetrachloroethene, benzene, 1,3,5-trimethylbenzene, 1,2,4-trimethylbenzene, o-xylene, and m,p-xylenes. The specific chemicals driving large (> 1) hazard scores were 1,2,4-trimethylbenzene, 1,3,5-trimethylbenzene, naphthalene, acetone, chloroform, benzene, m,p-xylenes, n-hexane, and o-xylene.

## 4 Discussion

In this study, we measured the indoor concentrations of VOCs in small business beauty salons serving primarily Latino and Spanish-speaking clientele in a low-income community in Tucson, Arizona. We demonstrated that real-time total VOC concentrations can vary over the work shift, while applying hair oil followed by thermal styling leads to peak exposures. However, the most significant variance in the mixed model was between shops. The most common specific VOC found in our study is 2-Propanol, often used in personal care products and other products used in salons. Specific VOC concentrations that were the most common include acetone, toluene, ethyl acetate, propene, and MEK; specific VOCs that were the most hazardous were naphthalene, chloroform, tetrachloroethene, benzene, 1,3,5-trimethylbenzene, 1,2,4-trimethylbenzene, o-xylene, and m,p-xylenes. Other studies investigating the air in beauty salon settings have found a combination of aromatics, esters, ketones, and terpenes (30). More data about workplace exposures in this setting are needed to develop a clearer picture of the fate and transport of these compounds. Additionally, robust workplace policies are needed that protect worker health instead of placing responsibility on them (31).

In this study, total VOCs aggregated over the activity-ventilation span ranged from less than the LOD of 1 ppb to a maximum of 76,892 ppb. Previous studies measuring total VOC concentration in beauty salons report values between 28 ppb and 5,248 ppb (12, 32). The maximum result from our studies is more elevated than those of these previous studies. In nail salon studies, higher total VOC concentrations (54,880 ppb) have been measured, like our maximum range (33). Further studies are needed to understand what activities and beauty products drive maximum values.

A key finding of this study was that there is more variation in VOC concentration between salons than within work shifts within salons. Most of the salons used central air conditioning for ventilation. We did not measure air exchange rates; therefore, differences in ventilation may be one of the drivers between these differences in salons. It needs to be further explored. Another key difference may be in the beauty product lines each salon utilizes. Each beauty product can have multiple variants within product lines with different chemical formulas. Small businesses in our study depend on various products and brands, unlike larger salons or salon spas that carry exclusive product lines. Choice of product(s) is primarily related to preference (client or beautician) and economics. In combination, varying products used in these small businesses tied to services can contribute to the differences in VOC concentrations between salons.

In some cases, individual salon owners and workers use different products and brands because workers often rent booths or chairs within the same salon. These salon workers are considered independent small businesses. Each small business can introduce different products and associated activities in this case. Also, beauty products generally have multiple variants within the product line with different chemical formulas. Sometimes, missing ingredient information can also be the case. Specialized products, such as Brazilian Blowout®, are tied to an exclusive service that requires specific beautician training. Yet, it can be the case that sometimes training protocols are not followed, and uncertified beauticians still use the product. These services’ uniqueness and associated products contribute to VOC variation between beauty salons. Therefore, choices made by each salon worker can affect the exposure of their colleagues to VOCs in the workplace, underscoring the importance of awareness in this context.

Styling products applied to the client’s hair in combination with thermal-styling can further explain the variation in total VOCs and the number of specific VOCs generated between salons. Heat introduced in the hair styling activity can accelerate the creation and volatilization of these compounds from the hair cuticle and scalp where they are applied initially. Thermal heat styling is accomplished by flat irons, hair dryers, hair processors, and styling combs, with potentially different heat elements. If a salon setting does not have adequate ventilation or air mixing, heat may stay in the indoor environment, potentially causing VOCs to combine further. For example, we did not see the VOC peaks from the hair oil (Figure 2) until after the beautician applied thermal styling. The safety data sheet for this specific product stated not to apply heat.

Tsigonia and colleagues (30) determined that the most significant variation in total VOC concentration depends on the use of products and associated characteristics, the number of services rendered, and the ventilation type in the salon space. In this and other studies, the total VOCs measured in beauty salons during a workday also showed significant variation (30, 34–36). Indoor air in beauty salon environments is a complex mixture of chemical ingredients, byproducts, vapors, and aerosols. Heat presented in this environment may increase the speed of chemical reactions. More controlled studies are needed to determine the interaction of the compounds and thermal styling in this environment.

Although there were no exceedances of occupational exposure guidelines, there were some exceedances of the U.S. EPA reference values. Some beauty salon business measurements resulted in hazard scores over the value of one. Settings with specific VOC concentrations exceeding the reference values or settings with hazard scores greater than one may be exposed to levels that may impact health. Exposure to high VOCs in beauty salons is expected to impact customers’ health less than workers because VOCs’ health effects are cumulative over time, and customers spend much less time in this high-VOC environment. Additionally, beauty salon workers handle diverse beauty products and have additional routes of exposure. In other studies, common VOCs detected include toluene, ethyl acetate, benzene, and acetone (30, 37, 38). The level of biomarkers in urine representing toluene exposure (N-acetyl-S-(benzyl)-L-cysteine) was reported by beauticians who used semi-permanent hair coloring formulations (39). Lamplugh and colleagues (40) found similar VOC compounds detected in our study as those in nail salons. The specific chemicals driving the hazard scores are reported in controlled studies focused on heating flame-retardant synthetic hair (37).

Other VOC sources, not associated with beauty or hair products, may also be present in indoor workplace air, contributing to the total VOCs present. A building and indoor space contains many natural and synthetic VOC sources that can add to this already burdened workplace environment (39). These additional sources, such as off-gassing paint or the use of cleaning products, add to the problem. These other VOC sources were not the focus of this study, so additional longitudinal measurements should be conducted to tease out the sources.

The percentage of specific VOCs detected by the Summa canister was low compared to the total VOCs detected by the PID. As there are likely 1000s of compounds present in the workplace, as expected, Summa canisters do not measure all the specific VOCs in the indoor air, and it is limited to only the 70 compounds that samples were analyzed for. The beauty salon setting is not a closed system. Chemical compounds generated in indoor air from different sources can impact general workers’ exposure, adding to their total chemical burden. Results from the Summa canister data analysis also determined the presence of other VOCs not in the U.S. EPA Air Method Toxic Organics −15, such as 2-methyl-1-butene. Compounds may mix and interact in the air in beauty salon environments, generating additional compounds. Also, thousands of chemicals are used in styling products, with many that still need reference values or analytical methods, so we cannot measure all of them. Therefore, the advantage of using the PID is to get an aggregate estimate of the total VOC exposures in the salon environment. Summa canisters cannot measure all the VOCs, but the data obtained is still helpful for future studies to identify ingredient generation rates and transport mechanisms.

Exposure scientists have also shown an increased chemical burden in beauty salons catering to women of color clientele (39–41). The literature outlines sociocultural and economic explanations that overtly drive high beauty product sales and associated activities. Racialized marketing and beauty standards perpetuate the purchase and use of products and services that contain more harmful chemicals than those targeted to their white counterparts (42, 43). Thus, participating beauticians, like in our study in salons mainly catering to clientele of color, may have a higher chemical burden in these salons (39, 40).

Previous studies have pointed to ventilation significantly influencing beauty salon air quality (35). In this study, ventilation was air-conditioning primarily, and we did not record air exchange rates, so we cannot formally assess the relationship between ventilation and total VOC concentrations. Another limitation is that we used the U.S. EPA reference values compared to occupation standards meant to protect the worker. Occupational standards are higher than environmental standards. Yet, new Occupational Safety and Health Administration (OSHA) standards, which are based on the feasibility of achieving a level in the worst segment(s) of industry, may not be sufficiently protective in sectors where exposures can be better controlled (44). None of the compounds exceeded the values of the OSHA or American Conference of Governmental Industrial Hygienists. Meanwhile, a key strength of the project is using the PID to understand the total VOCs in the air and data on beautician activity, business ventilation, and chemical inventory. Detailed data about a workplace setting can strengthen future studies regarding interventions.

The beauty salon is a complex and understudied occupational setting. Our findings confirm that applying styling products to the client’s hair and subsequent thermal-styling may explain part of the VOC variation between salons. While ventilation likely accounts for the main differences, beauty and styling products used in salons may also contribute to the VOC variation. The workplace environment (including indoor and outdoor areas surrounding the salon) may also add to the VOC variability between salons. The specific VOCs detected by the Summa canister are only a proportion of those that may exist in workplace air. The results of this study add to the evidence suggesting that salon workers can be exposed to steadily high concentrations of VOCs with periodic very high spikes. Following both the socioecological model of health and the hierarchy of controls, regulation, and inspection of industrial facilities that produce these products and precautionary development of product lines will significantly impact beauticians’ exposures to VOCs more than controls implemented on a shop-by-shop basis (31, 45). The results demonstrate that salon settings are incredibly diverse and poorly understood. To our knowledge, only a few studies have set out to assess workplace concentrations of VOCs in beauty salons with Latino workers focused on predominantly Spanish-speaking clients. Because of the unknown interaction of VOCs and the entirety of the variables involved, an expanded study design to capture more beauty salon spatial variability of VOCs during real-time would provide a more holistic perspective of what is happening.

## Data availability statement

The original contributions presented in the study are included in the article/Supplementary material, further inquiries can be directed to the corresponding author.

## Ethics statement

The studies involving humans were approved by Human Subjects Protection Program, University of Arizona. The studies were conducted in accordance with the local legislation and institutional requirements. The participants provided their written informed consent to participate in this study.

## Author contributions

DMR: Data interpretation, Writing – original draft, Writing – review & editing. SG: Formal analysis, Writing – original draft, Writing – review & editing. NL: Methodology, Writing – review & editing. CQ: Investigation, Writing – review & editing. MC: Investigation, Writing – review & editing. IC: Investigation, Writing – review & editing. FS: Investigation, Writing – review & editing. FC: Investigation, Writing – review & editing. EG: Investigation, Writing – review & editing. ET: Formal analysis, Writing – review & editing. RW: Investigation, Writing – review & editing. NL-G: Investigation, Writing – review & editing. MI: Investigation, Writing – review & editing. DB: Conceptualization, Funding acquisition, Project administration, Supervision, Writing – review & editing. AW: Conceptualization, Funding acquisition, Project administration, Supervision, Writing – review & editing. PB: Conceptualization, Funding acquisition, Project administration, Supervision, Writing – review & editing.
