# Supplementary material for: What a mix! Volatile organic compounds and worker exposure in small business beauty salons in Tucson, Arizona
Source: Front Public Health. 2023 Dec 18;11:1300291. doi: 10.3389/fpubh.2023.1300291 (PMC10757921; doi:10.3389/fpubh.2023.1300291)
Supplement: Supplementary file 1 [file Data_Sheet_1.PDF]

Supplementary Material

Chemical name	CAS #	Reference value ($\mu g/m^3$)	Source	HAPS International RfC (mg/m^3)	HAPS International RfC (ppm)	IH exposure limit (ppm)	IH exposure limit source
1,1-Dichloroethane	75-34-3			Not assessed under the IRIS	Not assessed under the IRIS	100	ACGIH TLV
1,1-Dichloroethene	75-35-4	200	RfC	0.20		5	ACGIH TLV
1,1,1-Trichloroethane	71-55-6	5000	RfC	5	0.916444271	10	ACGIH TLV
1,1,2-Trichloro-1,2,2-trifluoroethane	76-13-1	105000	RfD	Not assessed under the IRIS	Not assessed under the IRIS	1000	ACGIH TLV
1,1,2-Trichloroethane	79-00-5	6.25	IUR	Not assessed under the IRIS	Not assessed under the IRIS	10	ACGIH TLV
1,1,2,2-Tetrachloroethane	79-34-5	70	RfD	Information reviewed but value not estimated.	Not assessed under the IRIS	1	ACGIH TLV
1,2-Dibromoethane (EDB)	106-93-4	9	RfD	0.009	0.001171339	20	ACGIH TLV
1,2-Dichloro-1,1,2,2-tetrafluoroethane	76-14-2			Not assessed under the IRIS	Not assessed under the IRIS	1000	ACGIH TLV
1,2-Dichlorobenzene	95-50-1	315	RfD	Not assessed under the IRIS	Not assessed under the IRIS	25	ACGIH TLV
1,2-Dichloroethane	107-06-2	3.85	IUR	Not assessed under the IRIS	Not assessed under the IRIS	100	ACGIH TLV
1,2-Dichloropropane	78-87-5	4	RfC	0.004	0.000865632	10	ACGIH TLV
1,2,4-Trichlorobenzene	120-82-1	35	RfD	Not assessed under the IRIS	Not assessed under the IRIS	2	CDC
1,2,4-Trimethylbenzene	95-63-6	60	RfC	0.06	0.012205167	25	ACGIH TLV
1,3-Butadiene	106-99-0	2	RfC	0.002	0.000904015	2	ACGIH TLV

1,3-Dichlorobenzene	541-73-1			Not assessed under the IRIS	Not assessed under the IRIS	25	ACGIH TLV
1,3,5-Trimethylbenzene	108-67-8	60	RfC	0.06	0.012205167	25	ACGIH TLV
1,4-Dichlorobenzene	106-46-7	800	RfC	0.8	0.133063035	10	ACGIH TLV
1,4-Dioxane	123-91-1	30	RfC	0.03	0.008325199	20	ACGIH TLV
2-Butanone (MEK)	78-93-3	5000	RfC	5	1.695397118	200	ACGIH TLV
2-Hexanone	591-78-6	30	RfC	0.03	0.00732321	100	ACGIH TLV
2-Propanol	67-63-0			Not assessed under the IRIS	Not assessed under the IRIS	200	ACGIH TLV
2,2,4-Trimethylpentane	540-84-1			Not assessed under the IRIS	Not assessed under the IRIS	300	ACGIH TLV
4-Ethyltoluene	622-96-8			Not assessed under the IRIS	Not assessed under the IRIS	None listed	Not applicable
4-Methyl-2-pentanone (MIBK)	108-10-1	3000	RfC	3	0.732320963	20	ACGIH TLV
Acetone	67-64-1	3150	RfD	Information reviewed but value not estimated.	Information reviewed but value not estimated.	250	ACGIH TLV
Allyl chloride	107-05-1	1	RfC	0.001	0.000319512	1	ACGIH TLV
Benzene	71-43-2	30	RfC	0.03	0.009390122	0.5	ACGIH TLV
Benzyl chloride	100-44-7	2.06	OS	Information reviewed but value not estimated.	Not assessed under the IRIS	1	ACGIH TLV
Bromodichloromethane	75-27-4	70	RfD	Not assessed under the IRIS	Not assessed under the IRIS	None listed	Not applicable
Bromoform	75-25-2	90.9	IUR	Information reviewed but value not estimated.	Not assessed under the IRIS	0.5	ACGIH TLV
Bromomethane	74-83-9	5	RfC	5.00E-03	1.29E-03	200	ACGIH TLV

Carbon disulfide	75-15-0	700	RfC	0.7	0.224809867	1	ACGIH TLV
Carbon tetrachloride	56-23-5	100	RfC	0.1	0.015896132	5	ACGIH TLV
Chlorobenzene	108-90-7	70	RfD	Not assessed under the IRIS	Not assessed under the IRIS	10	ACGIH TLV
Chloroethane	75-00-3	10000	RfC	10	3.79	100	ACGIH TLV
Chloroform	67-66-3	4.35	RfD	Not assessed under the IRIS	Not assessed under the IRIS	10	ACGIH TLV
Chloromethane	74-87-3	90	RfC	0.09	0.043587204	100	ACGIH TLV
cis-1,2-Dichloroethene	156-59-2	7	RfD	Information reviewed but value not estimated.	Not assessed under the IRIS	200	ACGIH TLV
cis-1,3-Dichloropropene	10061-01-5	20	RfC	Not assessed under the IRIS	Not assessed under the IRIS	1	ACGIH TLV
Cyclohexane	110-82-7	6000	RfC	6	1.743066942	100	ACGIH TLV
Dibromochloromethane	124-48-1	70	RfD	Not assessed under the IRIS	Not assessed under the IRIS	None listed	Not applicable
Dichlorodifluoromethane	75-71-8	700	RfD	Not assessed under the IRIS	Not assessed under the IRIS	1000	ACGIH TLV
Ethanol	64-17-5			Not assessed under the IRIS	Not assessed under the IRIS	1000	NIOSH REL/OSHA PEL
Ethyl acetate	141-78-6	3150	RfD	Not assessed under the IRIS	Not assessed under the IRIS	400	ACGIH TLV
Ethylbenzene	100-41-4	1000	RfC	1	0.230295381	20	ACGIH TLV
Hexachlorobutadiene	87-68-3	4.55	IUR	Not assessed under the IRIS	Not assessed under the IRIS	0.02	ACGIH TLV
Isopropylbenzene	98-82-8	400	RfC	0.4	0.081367777	50	ACGIH TLV
m,p-Xylene	179601-23-1	100	RfC	0.1	0.023031274	100	ACGIH TLV
Methylcyclohexane	108-87-2			Not assessed under the IRIS	Not assessed under the IRIS	400	ACGIH TLV

Methylene Chloride	75-09-2	600	RfC	0.6	0.172736586	50	ACGIH TLV
Methyl tert-butyl ether	1634-04-4	3000	RfC	3		50	ACGIH TLV
n-Butylbenzene	104-51-8			Not assessed under the IRIS	Not assessed under the IRIS	10	NIOSH REL/OSHA PEL
n-Heptane	142-82-5			Not assessed under the IRIS	Not assessed under the IRIS	400	ACGIH TLV
n-Hexane	110-54-3	700	RfC	0.7	0.198600571	50	ACGIH TLV
n-Nonane	111-84-2			Not assessed under the IRIS	Not assessed under the IRIS	200	ACGIH TLV
n-Octane	111-65-9			Not assessed under the IRIS	Not assessed under the IRIS	300	ACGIH TLV
N-Propylbenzene	103-65-1			Not assessed under the IRIS	Not assessed under the IRIS	None listed	Not applicable
Naphthalene	91-20-3	3	RfC	0.003	0.000572269	10	ACGIH TLV
o-Xylene	95-47-6	100	RfC	0.1	0.023029104	100	ACGIH TLV
Propene	115-07-1			Not assessed under the IRIS	Not assessed under the IRIS	500	ACGIH TLV
sec-Butylbenzene	135-98-8			Not assessed under the IRIS	Not assessed under the IRIS	None listed	Not applicable
Styrene	100-42-5	1000	RfC	1	0.234753053	20	ACGIH TLV
tert-Butylbenzene	98-06-6			Not assessed under the IRIS	Not assessed under the IRIS	10	NIOSH REL/OSHA PEL
Tetrachloroethene	127-18-4	40	RfC	0.04	0.005897891	25	ACGIH TLV
Tetrahydrofuran	109-99-9	2000	RfC	2	0.678158847	50	ACGIH TLV
Toluene	108-88-3	5000	RfC	5	1.326770927	20	ACGIH TLV
trans-1,2-Dichloroethene	156-60-5	70	RfD	Not assessed under the IRIS	Not assessed under the IRIS	200	ACGIH TLV

trans-1,3-Dichloropropene	10061-02-6	20	RfC	Not assessed under the IRIS	Not assessed under the IRIS	1	ACGIH TLV
Trichloroethene	79-01-6	2	RfC	0.002	0.000372203	10	ACGIH TLV
Trichlorofluoromethane	75-69-4	1050	RfD	Not assessed under the IRIS	Not assessed under the IRIS	1000	ACGIH TLV
Vinyl acetate	108-05-4	200	RfC	0.2	0.056801022	10	ACGIH TLV
Vinyl bromide	593-60-2	3	RfC	0.003	0.000685835	0.5	ACGIH TLV
Vinyl chloride	75-01-4	100	RfC	0.1	0.039122504	1	ACGIH TLV

Versions and References for R and Packages

This section is included because knowing the versions of R and packages is essential for reproducible research and to give credit to the developers of the packages.

Versions of R and packages used in this report from the sessionInfo() command:

```
R version 4.3.1 (2023-06-16)
Platform: aarch64-apple-darwin20 (64-bit)
Running under: macOS Ventura 13.5


Matrix products: default
BLAS:   /Library/Frameworks/R.framework/Versions/4.3-arm64/Resources/lib/libRblas
.0.dylib
LAPACK: /Library/Frameworks/R.framework/Versions/4.3-arm64/Resources/lib/libRlapa
ck.dylib;  LAPACK version 3.11.0


locale:
[1] en_US.UTF-8/en_US.UTF-8/en_US.UTF-8/C/en_US.UTF-8/en_US.UTF-8


time zone: America/Phoenix
tzcode source: internal


attached base packages:
[1] stats     graphics  grDevices utils     datasets  methods   base


other attached packages:
 [1] report_0.5.7          forcats_1.0.0         stringr_1.5.0
```

```
 [4] dplyr_1.1.2           purrr_1.0.1           readr_2.1.4
 [7] tidyr_1.3.0           tibble_3.2.1          tidyverse_2.0.0
[10] ggforce_0.4.1         scales_1.2.1          viridis_0.6.4
[13] viridisLite_0.4.2     simplecolors_0.1.1    emmeans_1.8.7
[16] lmerTest_3.1-3        lme4_1.1-34           Matrix_1.6-0
[19] ggpubr_0.6.0          ggplot2_3.4.2         arsenal_3.6.3
[22] lubridate_1.9.2       kableExtra_1.3.4.9000 knitr_1.43

loaded via a namespace (and not attached):
 [1] tidyselect_1.2.0    farver_2.1.1        fastmap_1.1.1
 [4] tweenr_2.0.2        digest_0.6.33       timechange_0.2.0
 [7] estimability_1.4.1  lifecycle_1.0.3     magrittr_2.0.3
[10] compiler_4.3.1      rlang_1.1.1         sass_0.4.7
[13] tools_4.3.1         utf8_1.2.3          yaml_2.3.7
[16] ggsignif_0.6.4      labeling_0.4.2      bit_4.0.5
[19] xml2_1.3.5          abind_1.4-5         withr_2.5.0
[22] numDeriv_2016.8-1.1 grid_4.3.1          polyclip_1.10-4
[25] fansi_1.0.4         xtable_1.8-4        colorspace_2.1-0
[28] MASS_7.3-60         insight_0.19.3      cli_3.6.1
[31] mvtnorm_1.2-2       crayon_1.5.2        rmarkdown_2.23
[34] ragg_1.2.5          generics_0.1.3      rstudioapi_0.15.0
[37] httr_1.4.6          tzdb_0.4.0          minqa_1.2.5
[40] cachem_1.0.8        splines_4.3.1       parallel_4.3.1
[43] rvest_1.0.3         vctrs_0.6.3         boot_1.3-28.1
[46] webshot_0.5.5       jsonlite_1.8.7      carData_3.0-5
[49] bookdown_0.34       car_3.1-2           hms_1.1.3
[52] pbkrtest_0.5.2      bit64_4.0.5         rstatix_0.7.2
[55] systemfonts_1.0.4   jquerylib_0.1.4     glue_1.6.2
[58] nloptr_2.0.3        stringi_1.7.12      gtable_0.3.3
[61] munsell_0.5.0       pillar_1.9.0        htmltools_0.5.5
[64] R6_2.5.1            textshaping_0.3.6   vroom_1.6.3
[67] evaluate_0.21       lattice_0.21-8      highr_0.10
[70] backports_1.4.1     broom_1.0.5         bslib_0.5.0
[73] Rcpp_1.0.11         svglite_2.1.1       gridExtra_2.3
```

```
[76] nlme_3.1-162        xfun_0.39           pkgconfig_2.0.3
```

References for R and packages used in this report from the report::cite_packages() command:

- Bates D, Mächler M, Bolker B, Walker S (2015). "Fitting Linear Mixed-Effects Models Using lme4." *Journal of Statistical Software*, *67*(1), 1-48. doi:10.18637/jss.v067.i01 https://doi.org/10.18637/jss.v067.i01.
- Bates D, Maechler M, Jagan M (2023). *Matrix: Sparse and Dense Matrix Classes and Methods*. R package version 1.6-0, https://CRAN.R-project.org/package=Matrix.
- Garnier, Simon, Ross, Noam, Rudis, Robert, Camargo, Pedro A, Sciaini, Marco, Scherer, Cédric (2023). *viridis(Lite) - Colorblind-Friendly Color Maps for R*. doi:10.5281/zenodo.4678327 https://doi.org/10.5281/zenodo.4678327, viridisLite package version 0.4.2, https://sjmgarnier.github.io/viridis/.
- Garnier, Simon, Ross, Noam, Rudis, Robert, Camargo, Pedro A, Sciaini, Marco, Scherer, Cédric (2023). *viridis(Lite) - Colorblind-Friendly Color Maps for R*. doi:10.5281/zenodo.4679423 https://doi.org/10.5281/zenodo.4679423, viridis package version 0.6.4, https://sjmgarnier.github.io/viridis/.
- Grolemund G, Wickham H (2011). "Dates and Times Made Easy with lubridate." *Journal of Statistical Software*, *40*(3), 1-25. https://www.jstatsoft.org/v40/i03/.
- Heinzen E, Sinnwell J, Atkinson E, Gunderson T, Dougherty G (2021). *arsenal: An Arsenal of 'R' Functions for Large-Scale Statistical Summaries*. R package version 3.6.3, https://CRAN.R-project.org/package=arsenal.
- Kassambara A (2023). *ggpubr: 'ggplot2' Based Publication Ready Plots*. R package version 0.6.0, https://CRAN.R-project.org/package=ggpubr.
- Kuznetsova A, Brockhoff PB, Christensen RHB (2017). "lmerTest Package: Tests in Linear Mixed Effects Models." *Journal of Statistical Software*, *82*(13), 1-26. doi:10.18637/jss.v082.i13 https://doi.org/10.18637/jss.v082.i13.
- Lenth R (2023). *emmeans: Estimated Marginal Means, aka Least-Squares Means*. R package version 1.8.7, https://CRAN.R-project.org/package=emmeans.
- Makowski D, Lüdecke D, Patil I, Thériault R, Ben-Shachar M, Wiernik B (2023). "Automated Results Reporting as a Practical Tool to Improve Reproducibility and Methodological Best Practices Adoption." *CRAN*. https://easystats.github.io/report/.
- Müller K, Wickham H (2023). *tibble: Simple Data Frames*. R package version 3.2.1, https://CRAN.R-project.org/package=tibble.
- Pedersen T (2022). *ggforce: Accelerating 'ggplot2'*. R package version 0.4.1, https://CRAN.R-project.org/package=ggforce.
- R Core Team (2023). *R: A Language and Environment for Statistical Computing*. R Foundation for Statistical Computing, Vienna, Austria. https://www.R-project.org/.
- Riley J (2020). *simplecolors: Access Color Names Using a Standardized Nomenclature*. R package version 0.1.1, https://CRAN.R-project.org/package=simplecolors.
- Wickham H (2016). *ggplot2: Elegant Graphics for Data Analysis*. Springer-Verlag New York. ISBN 978-3-319-24277-4, https://ggplot2.tidyverse.org.
- Wickham H (2022). *stringr: Simple, Consistent Wrappers for Common String Operations*. R package version 1.5.0, https://CRAN.R-project.org/package=stringr.
- Wickham H (2023). *forcats: Tools for Working with Categorical Variables (Factors)*. R package version 1.0.0, https://CRAN.R-project.org/package=forcats.
- Wickham H, Averick M, Bryan J, Chang W, McGowan LD, François R, Grolemund G, Hayes A, Henry L, Hester J, Kuhn M, Pedersen TL, Miller E, Bache SM, Müller K, Ooms J,

Robinson D, Seidel DP, Spinu V, Takahashi K, Vaughan D, Wilke C, Woo K, Yutani H (2019). “Welcome to the tidyverse.” *Journal of Open Source Software*, *4*(43), 1686. doi:10.21105/joss.01686 https://doi.org/10.21105/joss.01686.

- Wickham H, François R, Henry L, Müller K, Vaughan D (2023). *dplyr: A Grammar of Data Manipulation*. R package version 1.1.2, https://CRAN.R-project.org/package=dplyr.
- Wickham H, Henry L (2023). *purrr: Functional Programming Tools*. R package version 1.0.1, https://CRAN.R-project.org/package=purrr.
- Wickham H, Hester J, Bryan J (2023). *readr: Read Rectangular Text Data*. R package version 2.1.4, https://CRAN.R-project.org/package=readr.
- Wickham H, Seidel D (2022). *scales: Scale Functions for Visualization*. R package version 1.2.1, https://CRAN.R-project.org/package=scales.
- Wickham H, Vaughan D, Girlich M (2023). *tidyr: Tidy Messy Data*. R package version 1.3.0, https://CRAN.R-project.org/package=tidyr.
- Xie Y (2023). *knitr: A General-Purpose Package for Dynamic Report Generation in R*. R package version 1.43, https://yihui.org/knitr/. Xie Y (2015). *Dynamic Documents with R and knitr*, 2nd edition. Chapman and Hall/CRC, Boca Raton, Florida. ISBN 978-1498716963, https://yihui.org/knitr/. Xie Y (2014). "knitr: A Comprehensive Tool for Reproducible Research in R." In Stodden V, Leisch F, Peng RD (eds.), *Implementing Reproducible Computational Research*. Chapman and Hall/CRC. ISBN 978-1466561595.
- Zhu H (2023). *kableExtra: Construct Complex Table with 'kable' and Pipe Syntax*. http://haozhu233.github.io/kableExtra/, https://github.com/haozhu233/kableExtra.
